# Breaking Cancer’s Momentum: CDK4/6 Inhibitors and the Promise of Combination Therapy

**DOI:** 10.3390/cancers17121941

**Published:** 2025-06-11

**Authors:** Yanbiao Liu, Seohyun Park, Yan Li

**Affiliations:** 1 School of Science, Auckland University of Technology, Auckland 1010, New Zealand; gww8665@autuni.ac.nz (Y.L.); stella.park@aut.ac.nz (S.P.); 2 Maurice Wilkins Centre, Auckland 1010, New Zealand

**Keywords:** CDK 4/6 inhibitor, cancer treatment, combination therapy

## Abstract

CDK4/6 inhibition represents a new generation of cancer therapies, targeting CDK4/6 complexes to induce cell cycle arrest in the G1 phase. These inhibitors have been widely used in combination with hormone receptor antagonists for treating ER+/HER2− breast cancer, achieving significant clinical success. Building on this progress, ongoing research explores novel combination therapies and expands the application of CDK4/6 inhibitors to other diseases. However, challenges remain, including variable cellular responses and the rapid development of drug resistance. Recent studies have uncovered new resistance mechanisms and their unexpected effects on cell metabolism, autophagy, and the tumor microenvironment beyond cell cycle arrest. This review provides a comprehensive overview of the mechanisms by which CDK4/6 inhibitors combat cancer and explores their potential for more effective and personalized treatment strategies.

## Simple Summary

CDK4/6 inhibition is a crucial therapy for targeting cell cycle dysregulation in cancer treatment. The combination of CDK4/6 inhibitors and hormone receptor antagonists has demonstrated remarkable clinical efficacy in treating ER+/HER2− breast cancer patients. Recent studies have uncovered new resistance mechanisms and their unexpected effects beyond cell cycle arrest. In this review, we describe the status of CDK4/6 inhibition in cancer treatment, focusing on mechanisms by which drug resistance develops and recent progress in combination therapies.

## 1. Introduction

Cell division is fundamental for the growth, development, and reproduction of living organisms. The cell cycle refers to a series of precisely regulated biological processes that occur between the completion of one cell division and the beginning of the next. In eukaryotic cells, the progression of the cell cycle is rigidly controlled by cyclins and cyclin-dependent kinases (CDKs). Cyclins are a large family of proteins expressed periodically, originally discovered by R. Timothy Hunt in 1982 [1]. They are classified into different types, each functioning at specific stages of the cell cycle by activating CDKs. CDKs comprise a large family of serine/threonine kinases with evolutionarily conserved regulation of the cell cycle in eukaryotic cells [2]. Each cyclin exists as an inactive monomer and forms a cyclin–CDK complex with its cognate CDK, which in turn phosphorylates substrates on serine and threonine residues to drive cell cycle progression. Such kinases are frequently overactivated in cancer cells to sustain proliferation, disrupting cell number homeostasis and normal tissue architecture.

**Figure 1:**
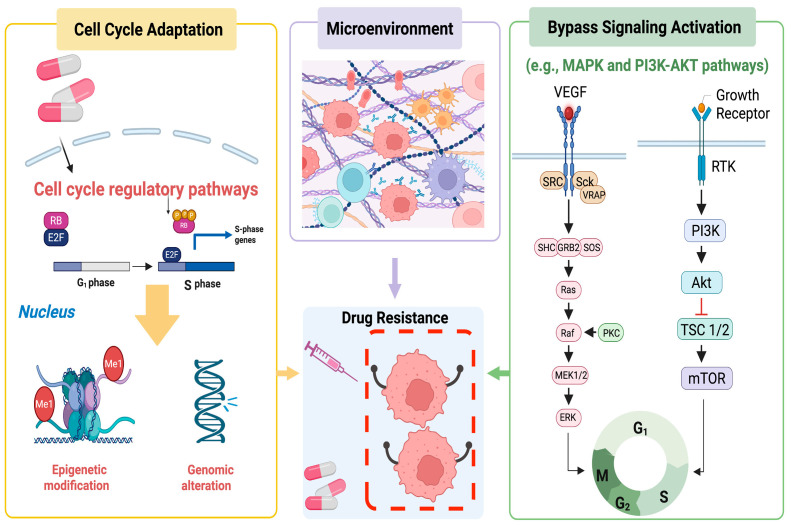
Mechanisms by which cancer cells develop drug resistance to CDK4/6 inhibitors (brief description). This figure was created in BioRender. Liu, Y. (2025) https://BioRender.com/kpcdv67 (accessed on 1 April 2025).

The inhibition of CDKs has emerged as a promising therapeutic strategy for cancer treatment, particularly following the introduction of broad-spectrum CDK inhibitors in the 1990s. Despite demonstrating effective tumour suppression activity, most first- and second-generation CDK inhibitors failed to progress through clinical trials due to poor selectivity and severe dose-limiting toxicities [3]. Subsequently, specific CDK inhibitors, particularly CDK4 and CDK6 inhibitors, minimised off-target toxicities while maintaining therapeutic efficacy. CDK4/6 inhibitors are now widely employed in breast cancer treatment and are being evaluated in various other malignancies. The synergistic application of CDK4/6 inhibitors and hormone receptor antagonists has demonstrated significant efficacy in treating estrogen receptor (ER)-positive and human epidermal growth factor receptor 2 (HER2)-negative breast cancer. However, the development of drug resistance remains a significant challenge. Cancer cells can develop resistance to CDK4/6 inhibitors through various mechanisms, including adaptive alterations in cell cycle regulatory pathways, reactive activation of bypass signalling pathways, and interactions with cancer-related immune cells in the microenvironment (Figure 1). Cancer cells resistant to CDK4/6 inhibitors exhibit significant differences from sensitive cells at the genomic, transcriptomic, and proteomic levels. Resistant cancer cells display augmented stem-like properties and heightened invasiveness, thereby increasing (elevating) the risk of treatment failure and recurrence [4,5]. To address the challenges posed by resistance, novel CDK4/6 inhibitors and more combination strategies are under development. Concurrently, recent studies have revealed the unexpected effects of CDK4/6 inhibitors on cancer cell senescence, metabolic state, and immune status, which may contribute to drug resistance and may guide future cancer therapeutics [6,7]. This review discusses the mechanisms by which CDK4/6 inhibitors combat cancer cells and highlights recent advancements in combination therapies.

## 2. Inhibition of CDK4/6 in Cancers

D-type cyclins are integral to the advancement of the cell cycle. There are three subtypes of D-type cyclins—cyclin D1, D2, and D3—under different tissue contexts. Different cyclins share functional similarities in activating CDK4/6, and cyclin D1 is more frequently dysregulated in human cancers [8,9]. Cyclin D1 was first identified as a potential oncogene in studies of B-lymphoid neoplasms [10]. Later, the mechanism of action of cyclin D1 became evident. Upon receiving external stimuli, growth factor receptors on the cell membrane activate cyclin D1, which then forms complexes with CDK4 and CDK6. Cyclin D1-CDK4/6 complexes gather in the cytoplasm and move into the nucleus. Upon phosphorylation by CDK-activating kinases (CAKs), cyclin D1-CDK4/6 complexes subsequently phosphorylate the retinoblastoma protein (Rb) at a singular phosphorylation site. In the early G1 phase, Rb exists in 14 distinct monophosphorylated forms, which together represent the “hypo-phosphorylated” state of Rb. In the hypo-phosphorylated state, Rb and related proteins p107 and p130 bind to the activation domain of E2F and inhibit its activity. E2F is a transcription factor that serves a pivotal role in regulating the transition of cells from the G1 phase to the S phase. By inactivating E2F, Rb hinders the G1 phase of development, thus preventing cell cycle progression and acting as a growth inhibitor. Phosphorylation of Rb by CDK4/6-cyclin D1 complexes reduces its ability to inhibit E2F transcription factors. Released E2F promotes transcription of the downstream genes, which are involved in DNA replication. Cells thus transition into the S phase. Concurrently, E2F promotes the expression and activation of cyclin E. Cyclin E associates with and activates cyclin-dependent kinase 2 (CDK2). The cyclin E-CDK2 complex facilitates the hyperphosphorylation of the Rb protein, thereby establishing a self-reinforcing feedback mechanism within this regulatory loop.

During oncogenesis, dysregulation of the cyclin D-CDK4/6-Rb-E2f pathway promotes aberrant cell cycle progression and uncontrolled proliferation of cancer cells. Therefore, targeted inhibition of CDK4/6 has emerged as a pivotal anticancer strategy. CDK4/6 inhibitors are small molecules characterised by pyrido [2,3-d] pyrimidine-7-one or 4-(pyrazole-4-yl)-pyrimidine scaffolds, with modifications designed to enhance selectivity for CDK4 and CDK6 over other kinases. These inhibitors induce G1 phase cell cycle arrest by preventing/impeding CDK4/6 activity. The U.S. FDA and the European Medicines Agency have approved three selective CDK4/6 inhibitors—palbociclib, abemaciclib, and ribociclib—for the treatment of ER+/HER2− breast cancer.

Palbociclib (PD-0332991), developed by Pfizer in 2001, demonstrated encouraging results in the phase III, multicenter, double-blind, randomised controlled trial (PALOMA-3). In patients with ER+/HER2− breast cancer whose disease had progressed after prior endocrine therapy, the combination of palbociclib and fulvestrant significantly extended the median progression-free survival (PFS) of these individuals [11]. Subsequently, Novartis and Eli Lilly and Company successively announced two additional CDK4/6 inhibitors: ribociclib (LEE011) and abemaciclib (LY2835219). In the MONALEESA and MONARCH clinical studies, ribociclib and abemaciclib, respectively, demonstrated promising efficacy outcomes [12,13,14,15,16,17]. Currently, all three agents are approved for use in both first-line and subsequent treatment settings for patients with metastatic ER+/HER2− breast cancer [18]. Ongoing research is actively exploring their application in additional cancer types and treatment strategies, including glioblastoma multiforme, mantle cell lymphoma, metastatic melanoma, and triple-negative breast cancer (TNBC) (NCT03090165) [19,20]. All three drugs, although formulated for oral administration, exhibit different pharmacokinetics and dosing strategies (Table 1). Despite a shared mechanism of cell cycle arrest, they exhibit distinct pharmacological characteristics and toxicity profiles. While both palbociclib and ribociclib exhibit cytotoxic effects in breast cancer cells, only palbociclib induces autophagy at higher doses. Abemaciclib has demonstrated a distinctive capability to traverse the blood–brain barrier and induces cancer cell death and G2 phase arrest compared with the two others. In terms of adverse effects, palbociclib is more likely to cause neutropenia compared to ribociclib and abemaciclib [21]. Ribociclib and abemaciclib are associated with more severe gastrointestinal toxicities [22]. Ribociclib also causes higher hepatic and respiratory toxicity as well as more severe cardiac damage compared to abemaciclib [23]. Despite these differences, no statistically significant differences in overall survival have been observed among palbociclib, ribociclib, and abemaciclib [24].

## 3. Development of Novel CDK4/6 Inhibitors

CDK4/6 inhibitors have achieved remarkable success in cancer therapy; however, the development of drug resistance during treatment remains inevitable. The underlying mechanisms include the loss of retinoblastoma (Rb) protein function and the activation of compensatory signalling pathways. In addition, dose-limiting toxicities, particularly hematologic and gastrointestinal adverse effects, continue to constrain broader clinical applications. These toxicities are partly attributed to the non-canonical functions of CDK4 and CDK6, which extend beyond cell cycle regulation and include roles in metabolism, stem cell maintenance, and immune modulation [25,26].

In response to these challenges, numerous next-generation dual CDK4/6 inhibitors have been developed to improve pharmacokinetics, minimise off-target effects, and enhance antitumor efficacy. GB491 (lerociclib), developed by G1 Therapeutics, displays increased potency against both CDK4 and CDK6, supporting continuous daily administration without treatment interruptions. In the phase III LEONARDA-1 trial, lerociclib combined with fulvestrant significantly improved progression-free survival (PFS) in patients with endocrine therapy-resistant breast cancer while maintaining a favourable safety profile [27].

Similarly, SHR6390 (dalpiciclib), a structurally optimised analogue of palbociclib and ribociclib, has demonstrated therapeutic potential in both ER+/HER2− breast cancer and head and neck mucosal melanoma, with improved binding properties and tolerability [28,29]. TQB3616 (bireociclib) has shown reduced gastrointestinal toxicity and enhanced antitumor activity in breast and lung cancer models, as confirmed in the BRIGHT-1 and BRIGHT-2 clinical trials [30]. Another dual inhibitor, trilaciclib, is primarily used to mitigate chemotherapy-induced myelosuppression but is also being evaluated for metastatic triple-negative breast cancer (TNBC) [31].

Despite these advances, dose-limiting hematologic toxicity, especially neutropenia resulting from CDK6 inhibition, remains a major obstacle to broader use. This has prompted a shift toward CDK4-specific inhibition to preserve antitumor efficacy while minimising systemic toxicity. Atirmociclib (PF-07220060) is a highly selective CDK4 inhibitor developed by Pfizer. Preclinical studies have demonstrated that atirmociclib provides more effective CDK4 suppression and stronger tumour growth inhibition in HR+/HER2− breast cancer while markedly reducing its impact on hematopoietic stem and progenitor cells compared to dual CDK4/6 inhibitors [32]. Theoretically, this improved therapeutic index permits higher dosing and supports the development of more potent synergistic combinations. Ongoing clinical trials, including NCT04557449, NCT06105632, and NCT06760637, are currently evaluating its use in combination with other therapeutic agents.

With the emergence of both optimised dual CDK4/6 inhibitors and CDK4-selective agents, therapeutic strategies are expanding. Increasing attention is now also being directed toward combination regimens that integrate these inhibitors with endocrine therapy, cytotoxic agents, targeted therapies, and immune checkpoint blockade to further enhance efficacy and overcome resistance.

## 4. CDK4/6 Inhibition and Chemotherapy

Traditional chemotherapy remains the cornerstone of cancer treatment. By either directly damaging the cellular genome or interfering with cell division, chemotherapeutic drugs effectively target rapidly dividing cancer cells [33,34]. The combination of emerging targeted therapies with traditional chemotherapy is a key strategy in cancer treatment to enhance efficacy and delay resistance [35,36].

Initially, the combination of CDK4/6 inhibitors with chemotherapy seemed counterintuitive due to their potentially antagonistic effects. CDK4/6 inhibitors induce cell cycle arrest in the G1 phase, resulting in diminished mitotic activity and a reduced temporal window for the action of chemotherapy agents, consequently undermining their efficacy. This incompatibility has been supported by multiple studies. For instance, in breast cancer models, the co-administration of palbociclib and carboplatin significantly reduced antitumor efficacy compared to carboplatin alone [37,38]. Similar antagonistic interactions have been documented in TNBC models with doxorubicin and in pancreatic cancer, where palbociclib impaired the effectiveness of mitotic agents and antimetabolites like gemcitabine and 5-FU [39]. In T-cell acute lymphoblastic leukaemia, ribociclib failed to augment the efficacy of multiple chemotherapeutic agents despite its potent single-agent activity [40].

These failures are now better understood mechanistically. By arresting cells in G1, CDK4/6 inhibitors limit the DNA damage response activated during S and G2/M phases, where many chemotherapeutics are effective. Moreover, CDK4/6 inhibition downregulates key components of the homologous recombination repair (HRR) pathway, further interfering with chemotherapeutic efficacy when administered concurrently or prior to cytotoxic agents.

However, recent research suggests this antagonism may be overcome through sequential scheduling. In high-grade serous ovarian cancer, sequential maintenance therapy with ribociclib following cisplatin administration exhibited a synergistic, lethal effect [41]. Similarly, in pancreatic cancer, sequential administration of palbociclib after taxanes was shown to suppress cancer recurrence [42]. These findings highlight the importance of temporal sequencing, allowing chemotherapy-induced DNA damage to occur prior to CDK4/6-mediated inhibition of repair pathways. Notably, this synergy is lost when CDK4/6 inhibitors precede or coincide with chemotherapy.

The variable interactions between CDK4/6 inhibitors and chemotherapeutic agents have also inspired another potential application for these inhibitors: the protection of hematopoietic stem cells and mitigation of chemotherapy-induced myelosuppression. This concept has been preliminarily realised with trilaciclib, a CDK4/6 inhibitor capable of inducing transient G1 arrest in hematopoietic stem and progenitor cells, thereby protecting them from chemotherapy-induced exhaustion in mice models [43]. This “pharmacologic quiescence” strategy illustrates the broader utility of CDK4/6 inhibition beyond direct tumour targeting.

## 5. CDK4/6 Inhibition and Other Targeted Therapies

Unlike traditional chemotherapy, which targets rapidly dividing cells, targeted therapy focuses on interfering with specific molecular features of cancer cells, such as mutated genes, overexpressed proteins, or abnormally activated signalling pathways. Targeted therapy offers greater precision and fewer side effects compared to chemotherapy, making it a promising direction for cancer treatment. Additionally, combining different targeted therapies can further enhance their anticancer efficacy and delay the development of drug resistance.

### 5.1. CDK4/6 Inhibition and Estrogen Receptor Antagonist Therapy

CDK4/6 inhibition and estrogen receptor (ER) antagonist therapy demonstrate strong synergistic effects in cancer treatment. Their combination can effectively suppress cancer cell proliferation and delay the onset of resistance [44]. Estrogen receptors (ERs), particularly ERα, act as transcription factors that promote the growth and survival of hormone-dependent tumours. In contrast, ERβ often exhibits tumour-suppressive properties, and its downregulation is associated with cancer progression. Antagonist [45]. Overexpression of ERβ can induce apoptosis of cancer cells and antagonise ERα activity through a proteasome-dependent degradation pathway [46,47]. Antagonist therapies, such as selective estrogen receptor modulators (SERMs) and degraders (SERDs), inhibit ERα signalling to suppress proliferation and induce apoptosis. Nonetheless, cancer cells can circumvent the suppression of ERα via CDK4/6-mediated reactivation of downstream proliferative signals, providing a rationale for combined therapy.

Following the approval by the Food and Drug Administration (FDA) of palbociclib in 2015 for the management of ER+/HER2− advanced recurrent breast cancer, CDK4/6 inhibitors—including palbociclib, ribociclib, and abemaciclib—have established themselves as standard therapeutic options for high-risk, early-stage postoperative ER+/HER2− breast cancer patients now in addition to those presenting with advanced disease [18]. In the PALOMA, MONALEESA, and MONARCH clinical trial series, the combination of CDK4/6 inhibitors with estrogen receptor antagonists significantly improved patient outcomes and demonstrated manageable side effects. This regimen has now become a standard treatment for advanced and metastatic ER+/HER2− breast cancer, which has been comprehensively summarised [44,48].

Meanwhile, the combination of CDK4/6 inhibition and estrogen receptor antagonist has shown potential in other hormone-dependent cancers. A combination of CDK 4/6 inhibition and estrogen receptor antagonists has shown promising results in phase II clinical trials on ER+ advanced endometrial cancer and low-grade serous ovarian carcinoma [49,50]. In addition, the combination of CDK inhibition and estrogen receptor antagonist has also demonstrated favourable effects in preclinical studies on prostate cancer. Concurrently, early-phase clinical trials are being conducted to assess the safety and feasibility of this combination therapy in the context of prostate cancer [51]. However, these successes remain preliminary. Unlike breast cancer, the molecular landscape, ER signalling dynamics, and resistance mechanisms in endometrial, ovarian, and prostate cancers are more heterogeneous and less well-characterised. Key gaps include a lack of predictive biomarkers, limited understanding of ER isoform expression patterns and unknowns about how CDK4/6 inhibition affects the tumour microenvironment in non-breast contexts. Further large-scale, mechanistically informed clinical trials are needed to validate efficacy, determine optimal treatment regimens, and identify the patient populations most likely to benefit from this combination strategy.

### 5.2. CDK4/6 Inhibition and RAS-MAPK Inhibition

**Figure 2:**
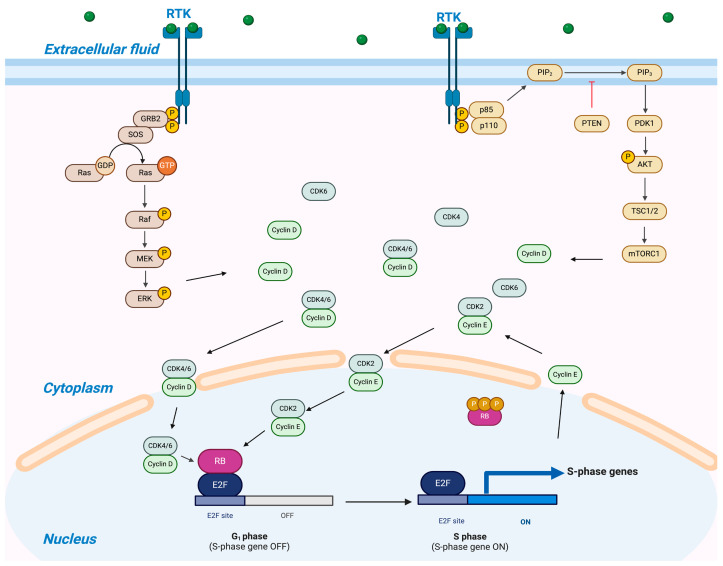
Crosstalk among CDK4/6-Rb-E2F, PI3K-AKT-mTOR, and RAS-MAPK in cancer cells. This figure was created in BioRender. Liu, Y. (2025) https://BioRender.com/g38n952 (accessed on 1 April 2025).

The RAS (rat sarcoma)-MAPK (mitogen-activated protein kinase) signalling cascade is a pivotal pathway through which extracellular cues regulate cell metabolism and proliferation [52]. One of its crucial downstream targets is cyclin D1, which promotes cell cycle progression via activation of cyclin D–CDK4/6 complexes (Figure 2). Oncogenic mutations in RAS, RAF, MEK, and ERK can result in sustained activation of the RAS/MAPK pathway, thus leading to sustained cyclin D1 expression and uncontrolled cell growth.

Inhibiting the RAS/MAPK pathway reduces cyclin D1 levels and indirectly suppresses CDK4/6 activity, while CDK4/6 inhibitors directly block cyclin D–CDK4/6 complexes. Thus, dual inhibition can synergistically arrest the cell cycle and reduce compensatory signalling.

Mutations of the RAS family are among the most frequent oncogenic drivers in human cancers. KRAS G12C is currently the only RAS mutation with FDA-approved inhibitors. Preclinical evidence supports that combining KRAS G12C inhibitors with CDK4/6 inhibitors could yield synergistic antitumor effects in NSCLC, colorectal, pancreatic, and oesophagal cancers, particularly when cell cycle control is compromised due to CDKN2A/B deletion or p16 inactivation [53,54]. These findings in pre-clinical studies provide a strong rationale for the clinical evaluation of this combination therapy. Ongoing trials like NCT05178888 and NCT05358249 aim to determine whether these synergistic effects could translate into clinical benefits for patients with KRAS G12C mutant solid tumours. Meanwhile, inhibitors targeting RAF, MEK, and ERK have demonstrated synergistic effects with CDK4/6 inhibitors in preclinical studies [55,56,57]. These combinations enhance cell cycle arrest and suppress compensatory oncogenic signalling, particularly in tumours driven by aberrant MAPK pathway activation. Early-phase clinical trials investigating these combination strategies have been initiated and are summarised in Table 2.

Moreover, an additional rationale supports the combined inhibition of the RAS/MAPK pathway and CDK4/6. Studies have found that the KRAS mutant cancer cells exhibit a high dependency on glutamine to support anabolic growth and proliferation [58]. This reprogramming creates a state of heightened metabolic dependency and reduced flexibility, rendering KRAS mutant cancer cells vulnerable to disruptions in key metabolic pathways. CDK4/6 inhibitors can exacerbate this vulnerability by halting cell cycle arrest and thus reducing biosynthetic activity. In metabolically stressed KRAS mutant cells, the combination of CDK4/6 inhibition with nutrient deprivation or metabolic pathway blockade has the potential to induce synthetic lethality [59]. This strategy exploits the intrinsic metabolic fragility of KRAS mutant cells and provides an expanded therapeutic option for overcoming drug-resistant cancers.

### 5.3. CDK4/6 Inhibition and PI3K-AKT-mTOR Inhibition

The PI3K-AKT-mTOR and CDK4/6-Rb pathways are two critical regulators of cell cycle progression and cancer proliferation. In cancer development, the PI3K-AKT-mTOR pathway is hyperactivated due to mutations in PI3K, AKT, or mTOR or loss of the tumour suppressor PTEN, contributing to uncontrolled growth and therapy resistance [60,61,62]. Importantly, this pathway interacts with the CDK4/6 axis: PI3K-AKT signalling promotes cyclin D1 expression, thereby activating CDK4/6 and driving Rb phosphorylation, which may underlie resistance to CDK4/6 inhibitors [63]. Conversely, active CDK4/6-Rb signalling has been shown to confer resistance to PI3K pathway inhibitors [64].

These reciprocal interactions provide a strong rationale for dual targeting. Preclinical studies in breast, pancreatic, and triple-negative breast cancer models have demonstrated that combined CDK4/6 and PI3K or mTOR inhibition can overcome monotherapy resistance, suppress proliferation, and induce immunogenic cell death [39,65,66]. Additionally, CDK4/6 inhibition may re-sensitise tumours to PI3K blockade [67]. These findings have prompted the early-phase clinical trials NCT03065062, NCT02732119, and NCT05563220 (Table 3), although larger randomised studies are still needed to evaluate long-term efficacy and safety.

Despite a strong biological rationale, several questions remain. The optimal sequencing or dosing strategies for dual inhibition are unclear, and predictive biomarkers to guide patient selection are lacking. Furthermore, the impact of dual inhibition on immune remodelling or metabolic adaptation, especially in non-breast tumours, has not been fully elucidated. These gaps represent key opportunities for future translational research.

### 5.4. CDK4/6 Inhibition and Receptor Tyrosine Kinase Inhibition

Receptor tyrosine kinases (RTKs) constitute a substantial family of cell surface receptors, encompassing 20 distinct families and a total of 58 receptors within the human genome [68]. They are also the primary upstream regulators of the PI3K-AKT-mTOR and RAS/MAPK signalling pathways mentioned above. Upon the binding of growth factors, including EGF, FGF, PDGF, and VEGF, to RTKs, the extracellular domain of the RTK experiences conformational alterations. This process subsequently results in the autophosphorylation of tyrosine residues within the intracellular domain. RTKs recruit and activate different molecules to activate multiple downstream signalling pathways, including the PI3K-AKT pathway, RAS-MAPK pathway, and JAK-STAT pathway.

Mutations or overexpressions of RTK genes result in persistent activation of intracellular signals, thus driving oncogenesis. Mutations of EGFR have been identified as a cause of non-small-cell lung cancer (NSCLC) [69], overexpression of HER2 is associated with breast cancer [70], and mutations of KIT (V-kit Hardy-Zuckerman 4 feline sarcoma viral oncogene homolog) are implicated in gastrointestinal stromal tumours [71]. Studies also suggest that the activity of RTKs is associated with cell sensitivity to CDK4/6 inhibitors [72,73]. Elevated RTK activity has been observed in lung cancer cells exhibiting resistance to CDK4/6 inhibitors. Lung cancer cells resistant to the CDK4/6 inhibitor palbociclib exhibit increased sensitivity to FGFR (fibroblast growth factor receptor) inhibition [74]. The concurrent inhibition of RTKs and CDK4/6 has exhibited a synergistic lethality in the context of cancer therapy. In HER2-positive breast cancer, the concomitant administration of the HER2 inhibitor lapatinib and the CDK4/6 inhibitor abemaciclib has demonstrated efficacy in suppressing breast cancer cells that exhibit resistance to lapatinib [75,76]. In EGFR mutant non-small-cell lung cancer, the CDK4/6 inhibitor Palbociclib can reverse the resistance to the EGFR inhibitor Osimertinib [77]. Table 4 summarises the current clinical studies investigating the combined treatment of CDK4/6 inhibitors and RTK inhibitors.

### 5.5. CDK4/6 Inhibition and Autophagy Inhibition

Autophagy is a degradation process in cells that removes unnecessary or dysfunctional components through lysosomes. It is essential for sustaining cellular homeostasis and can be triggered by several stresses. Research indicates that CDK4/6 inhibitors can stimulate autophagy in breast cancer cells by generating reactive oxygen species (ROS). In this context, autophagy acts as a cytoprotective mechanism that mitigates oxidative stress, thereby blunting the pro-senescent and pro-apoptotic effects of CDK4/6 inhibition. A recent study has shown that blocking autophagy can re-sensitise cancer cells to CDK4/6 inhibitors. Coadministration of autophagy inhibitors enhances the therapeutic efficacy of CDK4/6 inhibitors across a range of cancers, particularly in Rb-proficient and cyclin E-negative solid tumours [78].

The promising results in pre-clinical trials have led to clinical trials testing the efficacy of combining CDK4/6 inhibition and autophagy inhibition. Hydroxychloroquine (HCQ) is a synthetic derivative of chloroquine that belongs to the class of drugs called 4-aminoquinolines. HCQ serves as a potent inhibitor of autophagy. A phase I clinical trial (NCT03774472) published recently showed that HCQ is safe and well-tolerated and that adding HCQ can enhance the efficacy of low-dose Palbociclib (75 mg/day) and letrozole (2.5 mg/day) therapy in ER+/HER2− metastatic breast cancers [79]. A recent phase 1b/2, single-arm, open-label clinical study (NCT05953350) involved the recruitment of 29 patients with advanced HR+/HER2− breast cancer who had not responded to first-line Palbociclib therapy. The findings indicated that the combination of HCQ with the high-dose CDK4/6 inhibitor palbociclib (200 mg once a day) exhibited acceptable tolerability and satisfactory effectiveness [80].

As CDK4/6 inhibitor resistance becomes an increasingly recognised clinical problem, integrating autophagy modulation into treatment regimens could represent a next-generation strategy to restore and extend therapeutic responses. However, several challenges remain. Identifying predictive biomarkers will be crucial for patient stratification. Furthermore, the immunomodulatory effects of this combination therapy remain largely unexplored. The optimisation of dosing schedules to balance efficacy and toxicity also warrants further investigation.

## 6. CDK4/6 Inhibition and Immune Checkpoint Blockade

Over the past decade, immune checkpoint blockade (ICB) has revolutionised cancer therapy by reinvigorating the host immune system, particularly through PD-1/PD-L1 axis inhibition. PD-L1, expressed on tumour cells, binds PD-1 on cytotoxic T cells to suppress their activity and promote immune evasion. Targeting this axis with PD-L1/PD-1 inhibitors has achieved remarkable clinical success across several malignancies [81].

Emerging evidence suggests that CDK4/6 inhibitors can synergise with ICB by modulating both tumour cell immunogenicity and the microenvironment [82,83]. Mechanistically, CDK4/6 inhibitors regulate PD-L1 degradation via modulation of the CDK4–SPOP–E3 ligase axis. Inhibition of CDK4 downregulates SPOP, stabilising PD-L1 on the tumour surface [84]. This may prime tumour cells for enhanced recognition by T cells once checkpoint inhibition is applied.

Beyond PD-L1 expression, CDK4/6 inhibitors can reshape the tumour immune microenvironment. For instance, abemaciclib increases intratumoral CD8+ T cells and B cells by upregulating chemokines like CXCL10 and CXCL13 [83]. CDK4/6 inhibitors also promote antigen presentation by upregulating HLA class I molecules and inducing novel tumour-associated antigens, facilitating T-cell-mediated tumour clearance [85]. CDK4/6 inhibition could enhance the function of T cells. Studies have shown that short-term exposure to CDK4/6 inhibitors stimulates T-cell activation by suppressing NFAT transcription factors and improves memory T cells, a key factor in durable antitumor responses [86,87]. Moreover, CDK4/6 inhibition-induced senescence may contribute to innate immune activation, as senescent cells secrete chemokines (e.g., CCL2 and CXCL10) that recruit and activate NK cells [88].

In summary, current pre-clinical research demonstrates the significant potential of combining CDK4/6 inhibition with immune checkpoint inhibition in cancer treatment. Building on this, early-phase clinical trials are currently underway, which are summarised in Table 5.

However, translating these preclinical findings into consistent clinical benefit remains challenging. Appropriate patient selection is essential, as current evidence suggests that only tumours with functional retinoblastoma (Rb) protein, low cyclin E expression, and intact immune signalling pathways are likely to respond favourably. Additionally, the timing and sequencing of CDK4/6 inhibitors and ICB may critically influence therapeutic outcomes. However, the optimal scheduling strategy has yet to be fully elucidated. Furthermore, the development of predictive biomarkers is urgently needed to identify the patients most likely to benefit from this combinatorial approach. Despite these challenges, the combination of CDK4/6 inhibition and ICB holds significant potential for overcoming resistance to immunotherapy. CDK4/6 inhibitors are increasingly recognised not only for their cytostatic effects but also for their ability to modulate the tumour immune microenvironment, positioning them as promising agents in next-generation combination regimens.

## 7. Cell Cycle Arrest and Senescence Induced by CDK4/6 Inhibition

**Figure 3:**
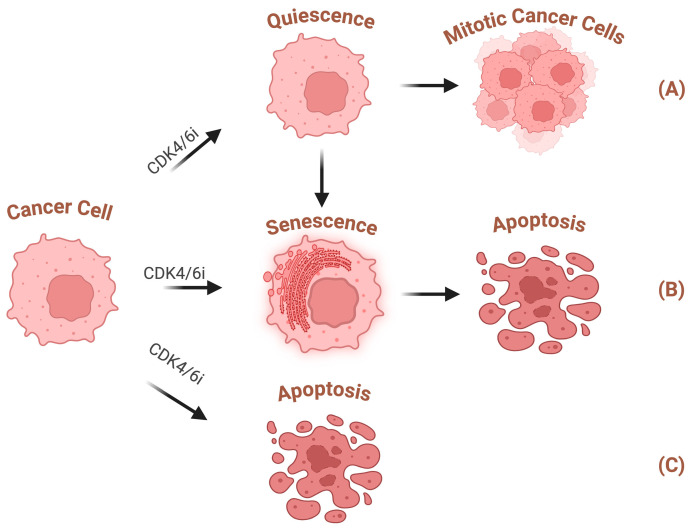
CDK4/6 inhibitors can elicit cancer cell cycle arrest. But this cell cycle arrest is not always permanent. (**A**) Quiescent cells can respond to mitotic signals, re-enter mitosis, or transition into senescent cells; (**B**) some other cells directly enter senescence state after CDK4/6 inhibition. Senescent cells can undergo apoptosis under senolytic drugs; (**C**) CDK4/6 inhibitors can induce apoptosis directly in certain conditions. This figure was created in BioRender. Liu, Y. (2025) https://BioRender.com/f12o917 (accessed on 10 April 2025).

CDK4/6 inhibitors induce cell cycle arrest at the G1 phase in Rb-proficient cancer cells. However, this arrest is context-dependent and not always permanent. In some cases, cells enter a reversible state known as quiescence, allowing them to resume proliferation once the inhibition is removed. In contrast, cells undergo senescence under certain conditions, a more stable and irreversible form of growth arrest (Figure 3). The choice between quiescence and senescence is influenced by several factors, including treatment duration, cellular stress levels, and the functional status of tumour suppressors [89,90]. Short-term CDK4/6 inhibition under low-stress conditions typically leads to reversible quiescence, while prolonged inhibition or high oncogenic or oxidative stress favours irreversible senescence.

Senescent cells exhibit specific characteristics, including enlarged cell size, enhanced granularity, elevated levels of senescence-associated β-galactosidase activity, and DNA damage foci [91]. Despite cell cycle arrest, they remain metabolically active and secrete a complex mix of factors known as the senescence-associated secretory phenotype (SASP), which reinforces their growth arrest and enhances the stemness and drug resistance of neighbouring cancer cells [92,93,94]. Interestingly, senescent cells exhibit increased sensitivity to certain therapeutic interventions despite their detrimental impact on the microenvironment. Studies have shown that SASP factors enhance immune clearance of cancer cells by recruiting innate immune cells and modulating adaptive immune responses [95,96]. And senescent lymphoma cells are more sensitive to glucose utilisation inhibition and autophagy inhibition compared to normal lymphoma cells [97]. Senescent cells are often detected following anti-cancer treatments, and therapy-induced senescence has been associated with improved patient prognosis [98,99].

Therefore, the distinction between quiescence and senescence is therapeutically significant. Understanding the factors that govern this cell fate decision is critical for improving the long-term efficacy of CDK4/6-targeted therapies. While contextual factors shape the overall cellular response, recent studies have identified specific molecular regulators that mediate the transition between these states.

**Figure 4:**
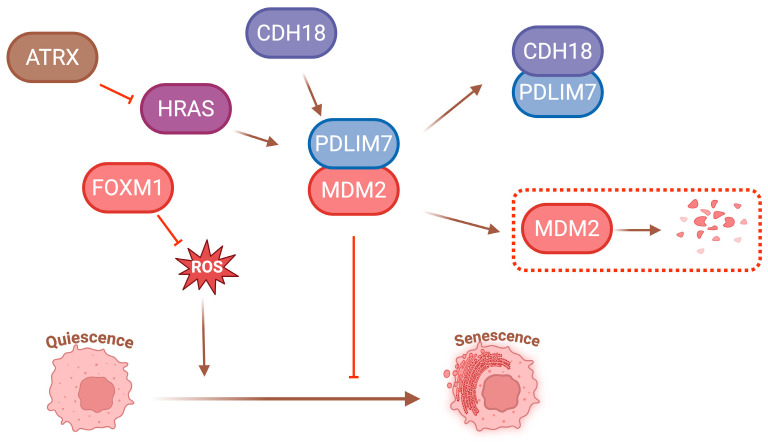
The transition from quiescence to senescence of cancer cells is influenced by MDM2 and reactive oxygen species (ROS). This figure was created in BioRender. Liu, Y. (2025) https://BioRender.com/f12o917 (accessed on 5 June 2025).

Mouse double minute 2 (MDM2), along with its related proteins, plays a crucial role in mediating the quiescence-senescence transition. MDM2 is an E3 ubiquitin ligase that targets p53 for ubiquitination and degradation, thereby suppressing the cell transition from quiescence to senescence. Notably, MDM2 can also undergo autoubiquitination, marking itself for proteasomal degradation. A cytoskeletal-associated scaffold protein, PDLIM7 (PDZ and LIM domain protein 7), binds to and stabilises MDM2, preventing its degradation. In well-differentiated and dedifferentiated liposarcoma (WD/DDLS) models, silencing PDLIM7 by shRNA promotes MDM2 degradation and facilitates the induction of senescence in quiescent cells [100]. Additionally, cadherin 18 (CDH18) has been shown to sequester PDLIM7 within a specific protein complex, thereby enhancing MDM2 degradation (Figure 4). As a result, targeting PDLIM7 and CDH18 has emerged as a promising strategy to potentiate the senescence-inducing effects of CDK4/6 inhibitors [100]. More research around MDM2 and cellular senescence transformation is currently underway. It has been revealed that MDM2 degradation is influenced by a chromatin remodelling protein ATRX [101,102]. ATRX plays a critical role in regulating gene expression and maintaining genomic stability by altering chromatin architecture. A recent study demonstrated that ATRX contributes to the induction of cellular senescence through the transcriptional repression of an oncogene HRAS (Figure 4) [103]. In a preclinical study, Vilgelm et al. showed that co-treatment with the CDK4/6 inhibitor ribociclib and the MDM2 antagonist nutlin-3a effectively overcame intrinsic resistance to CDK4/6 inhibition in melanoma patient-derived xenograft (PDX) models [104]. In addition, reactive oxygen species (ROS) have been implicated as an important mediator in the induction of senescence. CDK4/6 inhibition has been shown to elevate intracellular ROS levels by destabilising the transcription factor FOXM1, a key regulator of antioxidant gene expression. The resulting oxidative stress further amplifies senescence-associated signalling pathways, thereby reinforcing the senescent state [105].

Research on cellular senescence holds significant implications for improving cancer therapies. CDK4/6 inhibitors induce a state of growth arrest, pushing a subset of cancer cells into senescence rather than apoptosis. In this context, inhibitors that appear less effective against actively dividing cells may exert enhanced activity when administered after CDK4/6-mediated senescence induction. This sequential therapeutic strategy—targeting senescent cells following initial cell cycle arrest—opens the door for integrating senolytic agents, immune modulators, or metabolic inhibitors to eliminate senescent tumour cells selectively. Furthermore, identifying key proteins associated with the senescent phenotype may reveal novel therapeutic targets, enabling the development of rational drug combinations that enhance anti-tumour efficacy, lower required dosages, reduce systemic toxicity, and help overcome resistance mechanisms. Further research is warranted to establish greater confidence in supporting experimental and clinical correlative data and address key gaps in current knowledge.

## 8. Apoptosis Triggered by CDK4/6 Inhibition

Therapy-induced senescence is a promising strategy in cancer treatment. However, recent studies have revealed that senescence has both detrimental and beneficial effects on cancer treatment [106]. Senescence halts the cell cycle in cancer cells and prevents them from responding to growth-promoting signals. However, recent studies suggest that prolonged senescence, under certain conditions, can induce stem-cell-like properties in cancer cells. This includes increased expression of CD133 and Oct-4 and enhanced antioxidant defences, which may contribute to drug resistance and a higher risk of recurrence [107]. Given that senescent cells exhibit heightened sensitivity to specific cancer treatments compared to normal cells, a “double-hit” anti-cancer strategy could be developed. This approach involves inducing senescence in cancer cells with a drug, then selectively eliminating them using senolytic drugs (Figure 3). This strategy could treat previously difficult or even impossible cancers to cure [108].

One mechanism by which senescent cells evade apoptosis is the upregulation of anti-apoptotic B-cell lymphoma 2 (BCL-2) gene family proteins. Bcl-2 homology 3 (BH3) mimetics are a class of drugs that antagonise anti-apoptotic BCL-2 family proteins. ABT-199 (venetoclax) is a selective BCL-2 inhibitor that mimics the BH3 domain, binding with high affinity to BCL-2 and neutralising its pro-survival function. This disruption of BCL-2-mediated apoptosis inhibition leads to the induction of programmed cell death in cancer cells. A preclinical study showed that combining ABT-199/venetoclax with fulvestrant and palbociclib enhanced the treatment response by inducing apoptosis, intensifying the cell cycle arrest, and mitigating loss of Rb in ER-positive breast cancer. This study suggests that the combination of BCL2 inhibitors and CDK4/6 inhibitors is a promising double-hit anti-cancer strategy [109]. Currently, a phase Ib clinical trial is evaluating the efficiency of palbociclib, letrozole, and venetoclax in ER-positive and BCL-2-positive breast cancer patients (PALVEN, NCT03900884) [110].

Increased SA-β-Gal activity is a hallmark of senescent cells, as cellular senescence consistently leads to a rise in lysosomal mass. The increased β-galactosidase activity of senescent cells has also been used to design drugs with enhanced selectivity towards them. Nanoparticles coated with galactose-oligosaccharides can deliver anticancer drugs and selectively release them within senescent cells. Navitoclax is an inhibitor of BCL2 and BCL-xL. However, when used alone, severe off-target effects lead to significant thrombocytopenia, which limits its clinical application. Nanoparticle-encapsulated navitoclax Gal-NP (Nav) can specifically target senescent cells, effectively eliminating senescent cells while significantly reducing the systemic toxicity of navitoclax. In a pre-clinical study, Irene Galiana et al. used palbociclib to induce senescence in TNBC xenografted mice. The mice were then treated with Gal-NP (Nav). The results showed that, compared to the control group, the addition of Gal-NP reduced tumour size, prolonged the survival of the mice, and inhibited lung metastasis of TNBC [111].

Cardiac glycosides (CGs) are natural compounds used as Na^+^/K^+^-ATPase inhibitors in cardiology. Recent studies have found that CGs exhibit senolytic effects in cancer treatment [112,113]. This effect likely arises from elevated ion concentrations in senescent cells, which CGs counteract by inhibiting Na^+^/K^+^-ATPase and depolarising the plasma membrane [114]. It has been shown that ouabain and digitoxin can induce apoptosis in senescent lung cancer cells induced by palbociclib [113]. Digoxin can induce apoptosis in senescent melanoma cells induced by palbociclib [113]. However, in-depth preclinical studies and early clinical research on the combined efficacy of CGs and CDK4/6 inhibitors for cancer treatment are still lacking.

Targeted elimination of senescent cells induced by anticancer drugs is an imaginative and promising therapeutic strategy. In addition to the approaches we have already mentioned, current research has revealed more senolytic drugs, including flavonoid-based drugs, forkhead box o4 (FOXO4) mimetics, heat shock protein 90 (HSP90) chaperone protein inhibitors and their derivatives, bromodomain and extra terminal domain family protein degraders, as well as strategies to mobilize the immune system. Although research on combining these drugs with CDK4/6 inhibitors is still limited, senolytic drugs and CDK4/6 inhibitors have the potential to form a highly promising therapeutic combination in the future.

CDK4/6 inhibitors have traditionally been viewed as incapable of inducing cancer cell apoptosis on their own. Recent studies revealed that in certain conditions, CDK4/6 inhibitors can induce apoptosis through some non-conventional pathways (Figure 3). In a study on colon cancer, Thoms et al. revealed that CDK4 inhibition could trigger apoptosis via NF-κB signalling and nuclear transition of RelA [115]. In another study on non-small-cell lung cancer, Thangavel et al. discovered that palbociclib could induce apoptosis via inhibition of IAPs and up-regulation of RB-dependent pro-apoptotic factors [116]. One study demonstrated that in T-cell acute lymphoblastic leukaemia, palbociclib effectively inhibits the phosphorylation of glycolytic enzymes facilitated by CDK6. This inhibition ultimately results in an elevated concentration of ROS and promotes apoptosis [117]. This therapeutic effect is achieved through the inhibition of CDK6, relying on its unique functions and is independent of RB.

The anticancer mechanisms of CDK4/6 inhibitors are much more complex than originally thought. Exploring each mechanism further is promising, as it could enable more cancer patients to benefit from this treatment.

## 9. Metabolism Alterations Induced by CDK4/6 Inhibition

Since cell division is intimately related to cellular metabolism, it is not unexpected that by causing cell cycle arrest, CDK4/6 inhibitors might change the metabolism of cancer cells. Metabolism alterations enhance the adaptability of cancer cells, allowing them to survive in the presence of the CDK4/6 inhibitors, thereby promoting the development of drug resistance. In senescent cells induced by CDK4/6 inhibitor, researchers observed a significant increase in mitochondrial content and activity associated with oxidative response and inflammatory stress, which are often related to SASP [118].

Beyond inducing cellular senescence, recent studies have found that CDK4/6 inhibitors can also directly influence cellular metabolism through CDK4 and CDK6, which play important roles in cell metabolism. The phosphorylation of AMP-activated protein kinase alpha 2 (AMPKα2) mediated by CDK4 is correlated with an enhancement of glycolysis and a diminution of fatty acid oxidation in fibroblasts [119]. CDK4-mediated phosphorylation of general control of amino acid synthesis protein 5 is related to reduced glucose metabolism in hepatocytes [120]. The cyclin D3/CDK6 kinase complex phosphorylates 6-phosphofructokinase and pyruvate kinase M2, which are key steps in glycolysis [121]. Therefore, inhibition of CDK6 depletes nicotinamide adenine dinucleotide phosphate and glutathione, thereby increasing intracellular ROS levels. Correspondingly, researchers have found that in malignant pleural mesothelioma, palbociclib, whether used alone or in combination with PI3K/mTOR inhibitors, can reduce glucose uptake and glycolysis. This effect is probably achieved through inhibiting Rb/E2F/c-myc and PI3K/AKT/mTOR signalling pathways [122]. Similarly, the application of CDK4/6 inhibitors can also alter glucose metabolism and increase ROS levels in pancreatic ductal adenocarcinoma and TNBC [123,124].

In addition, CDK4/6 inhibitors affect the lipid metabolism of cancer cells. In ER+ breast cancer, researchers have found that the combination of palbociclib and endocrine therapy can induce disorders of lipid metabolism. Glutathione peroxidase 4 (GPX4), a selenium-dependent enzyme, plays a critical role in cellular antioxidant defence and lipid metabolism. The inhibition of GPX4 has produced synergistic fatal effects when combined with CDK4/6 inhibitors and endocrine treatment in ER+ breast cancer [118].

These metabolic alterations may act as a driver of resistance to therapy, but they also provide potential targets for new combination therapy strategies. Currently, research on anti-metabolic drugs, including inhibitors of glycolysis, fatty acid metabolism, and glutamine metabolism, is still underway and shows encouraging results [125,126]. Although comprehensive mechanistic studies and clinical research on the combination of CDK4/6 inhibitors and anti-metabolic drugs are still limited, the combination of CDK4/6 inhibition with metabolic blockade holds great potential as an effective treatment option for patients in the future.

## 10. Conclusions

CDK4/6 inhibitors exert stronger targeting capabilities and cause fewer or more manageable side effects compared to traditional chemotherapy. The combination of CDK4/6 inhibition with estrogen receptor antagonists has become a standard treatment for both early and advanced ER+/HER2− breast cancer patients. Furthermore, ongoing preclinical studies and early-phase clinical trials are dedicated to exploring the significant role of CDK4/6 inhibition in treating other cancer types. Encouragingly, in addition to their use in cancer treatment, CDK4/6 inhibitors are also being utilised to mitigate the side effects associated with traditional chemotherapy. Although the widespread application of this strategy still requires more robust scientific evidence and comprehensive evaluation, it is a bold and highly innovative approach. This review provides a comprehensive overview of the current advances in CDK4/6 inhibition in cancer therapy, with a focus on the associated signalling networks and combinatorial treatment strategies. Intracellular signalling regulation is intrinsically complex, and tumour cells frequently develop resistance to CDK4/6 inhibitors through compensatory activation of alternative proliferative pathways. Consequently, rationally designed combination therapies targeting parallel or intersecting signalling cascades have become a central strategy in overcoming therapeutic resistance and enhancing anti-tumour efficacy. Meanwhile, unexpected effects have emerged from the real-world use of CDK4/6 inhibitors, including their impact on cell metabolism, autophagy, and the tumour microenvironment. Although research in these areas remains in its early stages, emerging data have shown promising outcomes, laying a robust foundation for the future development of novel combination therapies. As preclinical studies and clinical trials advance, the use of CDK4/6 inhibitors—particularly in combination regimens—is anticipated to become increasingly widespread, potentially extending therapeutic benefits to a growing number of cancer patients.

## Figures and Tables

**Table 1:** Pharmacokinetics and dosing strategies of CDK4/6 inhibitors.

Name	Structure	Oral Bioavailability	IC_50_ in Cell-Free Assay (nM)	Time to Peak Concentration (h)	Half-Life (h)	Metabolism	Excretion	Standard Dose	Schedule
Palbociclib (PD0332991)	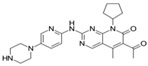	approximately 46%	CDK4 (11) CDK6 (15)	6–12	29	CYP3A4; SULT2A1	feces; urine	125 mg once daily	21 days on, 7 days off
Ribociclib (LEE011)	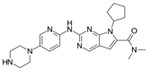	approximately 65.8%	CDK4 (10) CDK6 (39)	1–4	29.7–54.7	CYP3A4	feces; urine	600 mg once daily	21 days on, 7 days off
Abemaciclib (LY2835219)	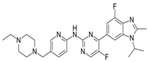	approximately 45%	CDK4 (2) CDK6 (9.9)	4–6	18.3	CYP3A4	feces; urine	150 mg twice daily	Continuous dosing

**Table 2:** Ongoing clinical trials on the combination therapy of CDK4/6 inhibitors and RAS-MAPK pathway inhibitors.

Identifier	CDK4/6 Inhibitor	RAS-MAPK Inhibitor	Conditions	Phase
NCT05358249	LEE011	MEK inhibitor: Trametinib; KRAS G12C inhibitor: JDQ443; EGFR inhibitor: Cetuximab	KRAS G12C mutant solid tumours	Ib/II
NCT05178888	Palbociclib	KRAS G12C inhibitor: MRTX849	Advanced solid tumour with KRAS G12C mutation	1/1b
NCT03170206	Palbociclib	MEK inhibitor: Binimetinib (MEK162)	Advanced KRAS mutant non-small-cell lung cancer	I
NCT02159066	LEE011	BRAF inhibitor: LGX818 and MEK inhibitor: MEK162	Locally advanced or metastatic BRAF V600 melanoma	II
NCT01543698	LEE011	BRAF inhibitor: LGX818 and MEK inhibitor: MEK162	BRAF V600-dependent advanced solid tumours	Ib/II
NCT03981614	Palbociclib	MEK inhibitor: Binimetinib (MEK162)	KRAS and NRAS mutant metastatic colorectal cancers	II
NCT01781572	LEE011	MEK inhibitor: Binimetinib (MEK162)	NRAS mutant melanoma	Ib/II
NCT03454035	Palbociclib	ERK1/2 inhibitor: Ulixertinib (BVD-523)	Pancreatic cancer and metastatic melanoma	I
NCT03132454	Palbociclib	Multi-kinase inhibitor: Sorafenib	Relapsed and refractory leukemias	I
NCT02065063	Palbociclib	MEK inhibitor: Trametinib	Solid tumours	I
NCT02022982	Palbociclib	MEK inhibitor PD-0325901	KRAS mutant non-small-cell lung cancer and other solid tumours	I

**Table 3:** Ongoing clinical trials on the combination therapy of CDK4/6 inhibitors and PI3K-AKT-mTOR pathway inhibitors.

Identifier	CDK4/6 Inhibitor	PI3K-AKT-mTOR Inhibitor	Conditions	Phase
NCT05563220	Palbociclib or Ribociclib or Abemaciclib	PI3K inhibitor: Alpelisib or mTOR inhibitor: Everolimus or AKT inhibitor: Capivasertib	Metastatic breast cancer	I/II
NCT03065062	Palbociclib	Dual PI3K/mTOR inhibitor: Gedatolisib	Advanced squamous cell lung, pancreatic, head and neck, and other solid tumours	I
NCT02626507	Palbociclib	Dual PI3K/mTOR inhibitor: Gedatolisib	ER+/HER2− breast cancer	I
NCT03006172	Palbociclib	PI3Kα inhibitor: Inavolisib	Locally advanced or metastatic PIK3CA mutant breast cancer	I
NCT02985125	LEE011	mTOR inhibitor: Everolimus	Metastatic pancreatic adenocarcinoma	I/II
NCT03008408	Ribociclib	mTOR inhibitor: Everolimus	Advanced or recurrent endometrial carcinoma	II
NCT03114527	Ribociclib	mTOR inhibitor: Everolimus	Advanced dedifferentiated liposarcoma (DDL) and leiomyosarcoma (LMS)	II
NCT01872260	LEE011	PI3Kα inhibitor: Alpelisib (BYL719)	ER+/HER2− locally advanced or metastatic breast cancer	I/II

**Table 4:** Ongoing clinical trials on the combination therapy of CDK4/6 inhibitors and RTK inhibitors.

Identifier	CDK4/6 Inhibitor	RTK Inhibitor	Conditions	Phase
NCT03304080	Palbociclib	HER2 inhibitor: trastuzumab and pertuzumab	HR-positive, HER2-positive metastatic breast cancer	I/II
NCT03132454	Palbociclib	Multikinase RTK inhibitor: Sorafenib	Relapsed and refractory leukemias	I

**Table 5:** Ongoing clinical trials on the combination therapy of CDK4/6 inhibitors and immune checkpoint inhibitors.

Identifier	CDK4/6 Inhibitor	ICI Inhibitor	Conditions	Phase
NCT02791334	Abemaciclib	LY3300054	HR+/HER2− breast cancer	I
NCT04118036	Abemaciclib	Pembrolizumab	Recurrent glioblastoma	II
NCT02778685	Palbociclib	Pembrolizumab	Newly diagnosed metastatic stage IV estrogen receptor-positive breast cancer	II
NCT04075604	Palbociclib	Nivolumab	ER+/HER2− breast cancer	II
NCT02779751	Abemaciclib	Pembrolizumab	Non-small-cell lung cancer or breast cancer	I
NCT03147287	Palbociclib	Avelumab	HR+/HER2− breast cancer	II
NCT03280563	Abemaciclib	Atezolizumab	Hormone receptor-positive, HER2-negative breast cancer	Ib/II
NCT04272645	Abemaciclib	Atezolizumab	Metastatic castration-resistant prostate cancer	II
NCT04220892	Abemaciclib	Pembrolizumab	High-grade glioma	I
NCT03997448	Abemaciclib	Pembrolizumab	Advanced gastric, gastroesophageal junction, esophageal adenocarcinoma	II
NCT04088032	Abemaciclib	Durvalumab	Locally advanced hormone receptor-positive breast cancer	I
NCT03294694	Ribociclib	PDR001	Metastatic hormone receptor-positive breast cancer and metastatic ovarian cancer	Is
NCT04360941	Palbociclib	Avelumab	Metastatic AR+ triple-negative breast cancer	Ib

## Abbreviations

ER	Estrogen receptor
HER2	Human epidermal growth factor receptor 2
CDK	Cyclin-dependent kinase
CAK	CDK-activating kinase
PFS	Progression-free survival
TNBC	Triple-negative breast cancer
FDA	Food and Drug Administration
RAS	Rat sarcoma
MAPK	Mitogen-activated protein kinase
RAF	Rapidly accelerated fibrosarcoma
MEK	Mitogen-activated protein kinase
ERK	Extracellular signal-regulated kinase
PDX	Patient-derived xenograft
PI3K	Phosphoinositide 3-kinase
PIP2	Phosphatidylinositol (4,5)-bisphosphate
PIP3	Phosphatidylinositol (3,4,5)-trisphosphate
PDK	Phosphoinositide-dependent kinase
MTORC2	Mechanistic target of rapamycin complex 2
PTEN	Phosphatase and tensin homolog
RTK	Receptor tyrosine kinase
JAK-STAT	Janus kinase-signal transducer and activator of transcription
FGFR	Fibroblast growth factor receptor
EGFR	Epidermal growth factor receptor
ROS	Reactive oxygen species
HCQ	Hydroxychloroquine
ICB	Immune checkpoint blockade
PD-1	Programmed cell death protein 1
PD-L1	Programmed cell death-ligand 1
SPOP	Speckle-type POZ protein
CXCL	C-X-C motif chemokine ligand
NFAT	Nuclear factor of activated T cell
HLA	Human leukocyte antigen
MHC	Major histocompatibility complex
NK cell	Natural killer cell
SASP	Senescence-associated secretory phenotype
MDM2	Mouse double minute 2
WD/DDLS	Well-differentiated and dedifferentiated liposarcoma
PDLIM7	PDZ and LIM domain 7
CDH18	Cadherin 18
FOXM1	Forkhead box M1
BH3	Bcl-2 homology 3
BCL-2	B-cell lymphoma 2
CG	Cardiac glycoside
FOXO4	Forkhead box o4
HSP90	Heat shock protein 90
AMPKα2	AMP-activated protein kinase alpha 2
GPX4	Glutathione peroxidase 4
Rb	Retinoblastoma protein
